# Prevalence of exocrine pancreatic insufficiency at 12 months after acute pancreatitis: a prospective, multicentre, longitudinal cohort study

**DOI:** 10.1016/j.eclinm.2024.102774

**Published:** 2024-08-02

**Authors:** Anna Evans Phillips, Joseph Bejjani, Stacey Culp, Jennifer Chennat, Peter J. Lee, Jorge D. Machicado, Vikesh K. Singh, Elham Afghani, Mitchell L. Ramsey, Pedram Paragomi, Kimberly Stello, Melica Nikahd, Phil A. Hart, Georgios I. Papachristou

**Affiliations:** a Division of Gastroenterology, Hepatology, and Nutrition, Department of Medicine, University of Pittsburgh School of Medicine, Pittsburgh, PA, USA; b Division of Gastroenterology, Hepatology, and Nutrition, The Ohio State University, Wexner Medical Center, Columbus, OH, USA; c Center for Biostatistics, Department of Biomedical Informatics, The Ohio State University Wexner Medical Center, Columbus, OH, USA; d Division of Gastroenterology and Hepatology, University of Michigan, Ann Arbor, MI, USA; e Division of Gastroenterology and Hepatology, Department of Medicine, Johns Hopkins Medical Institutions, Baltimore, MD, USA

**Keywords:** Exocrine pancreatic insufficiency (or pancreatic exocrine insufficiency), Exocrine pancreatic dysfunction, Acute pancreatitis, Malabsorption

## Abstract

**Background:**

Exocrine Pancreatic insufficiency (EPI) occurs following acute pancreatitis (AP) at variably reported rates and with unclear recovery timeline. The aim of this study was to establish the prevalence and predictors of EPI at 12 months after AP in a prospective cohort.

**Methods:**

In this prospective, multicentre, longitudinal cohort study, adult participants (≥18 years) admitted to the hospital with an AP attack (defined by Revised Atlanta Classification) were enrolled in a United States multi-centre longitudinal cohort (Sites: The Ohio State University, University of Pittsburgh, and Johns Hopkins University). Patients were excluded if they had pancreatic cancer, chronic pancreatitis, or malabsorptive disease (including previously diagnosed EPI). Participant data was obtained by interview and by review of the electronic medical record. EPI was assessed by stool fecal elastase (FE-1) levels collected at baseline, 3 months, and 12 months (primary endpoint). EPI was defined by FE-1 <200 μg/g; severe FE-1 level ≤100 μg/g; mild FE-1 101–200 μg/g. Multivariable logistic regression was used to identify predictors of EPI at 12 months. This study is registered with ClinicalTrials.gov, NCT03063398.

**Findings:**

EPI was observed in 29 (34.1%) of the 85 participants [44 (51.8%) male, mean age 54.7 ± 14.1 years] who provided stool samples at 12 months. For the study overall, participants were recruited between June 22, 2017 and October 18, 2021. A total of 5794 individuals were screened, 311 of whom were eligible for the study. 112 participants provided stool samples at baseline, 79 completed stool samples at 3 months, and 85 completed samples at 12 months. 64 participants included samples at all 3 timepoints. In univariable analysis, factors significantly associated with EPI at 12 months included recurrent (versus index) AP, pre-existing diabetes, alcohol, and idiopathic etiologies, and increasing severity of AP. In multivariable analysis, the odds of having EPI at 12 months increased 4-fold with idiopathic AP etiology (Odds Ratio 4.095, 95% Confidence Interval [CI] 1.418, 11.826), and 3-fold with moderately severe or severe AP (Odds Ratio 3.166, 95% CI 1.156, 8.670), and baseline diabetes mellitus (Odds Ratio 3.217, 95% CI 1.113, 9.298). Even individuals with an index mild attack of AP (n = 39) developed severe EPI at 12 months (prevalence 12.8%).

**Interpretation:**

EPI as diagnosed by FE-1 is present in over one third of prospectively assessed patients at 12 months post-AP. Since EPI develops in patients with mild AP, investigations are needed to understand the mechanisms of injury and identify methods for tailored screening.

**Funding:**

This study was supported by an Investigator Initiated Research Grant from 10.13039/100006483AbbVie, Inc.

**Research in context**

**Evidence before this study**

We searched PubMed for studies published until January 1, 2017, using search times including exocrine pancreatic insufficiency (EPI), exocrine pancreatic dysfunction (EPD), acute pancreatitis (AP), and malabsorption in abstract, title or MESH headings, and additionally reviewed references listed in papers of interest that were identified. Previous to this study, it was noted that EPI occurs following acute pancreatitis (AP) at variably reported rates, with unclear recovery timeline, and with inconsistently reported associations. In addition to this, EPI has been diagnosed with varying techniques. Particularly lacking were prospective data and standardised diagnostic technique for EPI after AP. Identifying patients with EPI after AP and associated risk factors for its development after AP represent the first steps in improving care for this population of patients.

**Added value of this study**

To our knowledge, this is the largest prospective study of prevalence and predictors of EPI at 12 months after AP. The results indicate that EPI, as diagnosed by fecal elastase-1, occurs after AP in a large portion of the population: this may have benefit in convincing caretakers of this population to screen for the condition and treat it effectively. Particular attention may be paid to those patients with idiopathic AP etiology, moderately severe or severe disease, or diabetes at baseline, all of which place patients with AP at higher risk of developing EPI.

**Implications of all the available evidence**

EPI may be present in over one third of prospectively assessed patients at 12 months post-AP. While specific subpopulations may have identified clinical risk factors, it will remain important to have a low threshold for testing and treatment as there remains much to learn about mechanisms leading to EPI after AP.

## Introduction

Exocrine pancreatic insufficiency (EPI) is a deficiency in the production and/or secretion of pancreatic enzymes which can lead to maldigestion and malabsorption of nutrients.^1^ The diagnosis has long been associated with chronic pancreatitis (CP) in the setting of end-stage disease with extensive fibrosis, acinar atrophy, pancreatic ductal obstruction, and the inability of the gland to either make or effectively secrete pancreatic digestive enzymes.^2^ In contrast, the natural history of EPI in the setting of acute pancreatitis (AP) is less well-defined, including its prevalence during or after an AP episode.^3^^,^^4^

Previously thought to be a self-limited phenomenon of acute inflammation that resolved on its own in the majority of cases (except in those with pancreatic necrosis and evidence of systemic complications), AP is now understood to have multiple metabolic sequelae, including EPI and diabetes.^5^, ^6^, ^7^, ^8^ The time course of these developments, and their pathophysiology currently remain poorly studied. The pooled prevalence of EPI by systematic review and meta-analysis following AP at various timepoints has been estimated between 27% and 36%, but these estimates are hindered by the variable methods of diagnosing EPI, retrospective nature of studies, and often short-term followup.^3^^,^^4^ Predictors of EPI at these various timepoints have included severe AP and pancreatic necrosis.^3^^,^^4^ Little is known about the risk of EPI in the first 12 months after AP attack, when the period of recovery from acute inflammation is likely to be ongoing.

The clinical importance of EPI after AP also remains unknown. In CP, untreated EPI over the long term can lead to maldigestion, malabsorption, and nutritional deficiencies that result in metabolic bone disease, sarcopenia, and even increased mortality.^9^^,^^10^ In the absence of a clear understanding of the natural history surrounding this diagnosis, an accurate understanding of the potential for treatment and opportunity to improve long-term AP outcomes remains elusive. Underestimating this potentially key complication of AP however does so at the potential peril of patients who may be suffering from nutritional deficiencies, abdominal symptoms secondary to EPI, or impaired quality of life (QOL) from a readily treatable condition.^11^^,^^12^

There is a lack of prospective data on the burden of EPI after AP in predetermined time intervals, including a clear understanding of associated features that may predict its occurrence. The aim of this study is to establish the burden and predictors of EPI at 12 months after AP in a large, prospective cohort of patients with AP.

## Methods

### Study design and participants

This was a prospective, multicentre, longitudinal cohort study (Post-Acute Pancreatitis Pancreatic Exocrine Insufficiency [PAPPEI]) conducted between 2017 and 2021; methods have previously been published elsewhere.^13^ The registered study (clinicaltrials.gov NCT03063398) enrolled participants at three tertiary institutions: the University of Pittsburgh Medical Center, The Ohio State University Wexner Medical Center, and Johns Hopkins University Medical Center. Ethical approval was obtained prior to enrollment of participants at all three centres. This study adheres to the appropriate Enhancing the Quality and Transparency of Health Research (EQUATOR) reporting guidelines (Strengthening the Reporting of Observational Studies in Epidemiology [STROBE]).

Adult (≥18 years of age) hospital inpatients who met diagnostic criteria for AP according to the Revised Atlanta Criteria were assessed for eligibility.^14^ Elevated lipase level alerts were set up to be sent from clinical laboratories to research staff through the electronic medical health record at each institution as a means of alerting study staff to potentially eligible patients. Severity of AP was also classified according to the Revised Atlanta Criteria.^14^ Participants were excluded if they had pre-existing diagnosis of chronic pancreatitis (CP) including evidence of pancreatic calcifications on imaging, or pre-existing diagnosis or history of pancreatic cancer. Participants were also excluded if they had an existing diagnosis of EPI prior to study enrollment, gastroparesis, cystic fibrosis, or other identifiable cause of altered small intestinal absorption including history of gastric bypass, pancreatic surgery, or a small bowel condition such as celiac disease or inflammatory bowel disease. Informed consent was provided prior to any study procedures. Participants included in the current analysis were those who completed stool samples at their 12-month follow-up timepoint.

### Clinical data collection

Case report forms (CRFs) were completed during hospitalization by the clinical research coordinator (CRC) in conjunction with the study participant and included data on demographics and medical history. Information from the Electronic Medical Record (EMR) was also obtained for AP etiology, history of previous AP episodes, prior EPI history or diagnosis, and past medical history. Vital signs and imaging data were also obtained from the EMR by the CRC with input from site investigators where physician expertise was required. Additional CRFs were completed at 3 and 12 months during an in person or virtual research visit.

### Fecal elastase testing

Stool samples were collected at three time points for each participant: baseline (window: day of enrollment to up to 30 days), at 3 months (window 2–5 months), and at 12-months (window 10–16 months) after enrollment. Presence of EPI was assessed using fecal elastase-1 (FE-1) levels from stool samples. FE-1 was measured via enzyme-linked immunosorbent assay quantifying CELA2/CELA3 isoforms (EasySampler® Stool Collection Kit, ALPCO Inc., Salem, NH). All manufacturer instructions were followed. To minimise false-positive results, participants were asked to collect formed stool samples if able.^15^ FE-1 > 200 μg/g stool was considered normal, and EPI was operationalised as FE-1 ≤ 200 μg/g stool for analysis; values 100 < FE-1 level ≤200 μg/g stool were considered mild EPI, and FE-1 level ≤100 μg/g stool was considered severe EPI. Results were reported to the participant's clinical pancreatology provider including gastroenterologist or primary medical provider to follow up regarding potential treatment with pancreatic enzyme replacement therapy (PERT).

### Statistical analysis

Demographics, clinical characteristics, and disease features for those with and without EPI at 12 months were compared using independent sample t-test for continuous variable and chi-squared test for categorical variables (univariable analysis). Missing data were not assessed. Demographics, clinical characteristics, and disease features at baseline with a statistically significant relationship (α<0.05) with EPI at 12 months in the univariable analysis were entered simultaneously as covariates in a multivariable logistic regression analysis to calculate adjusted odds ratios (ORs) for the prediction of EPI at 12 months. The significant predictors from this model were entered simultaneously to construct a final parsimonious logistic regression model. The results from multivariable analysis are presented as ORs with 95% CI. Prevalence of EPI was calculated for all available participants at baseline, 3 months, and 12 months by EPI severity. Prevalence of EPI at 12 months was further described by subsets based on AP history and AP severity. The trajectory of FE-1 was evaluated for participants for whom stool samples were available at all three time points using a Sankey diagram. All analyses were performed using R version 4.1.2 (R Foundation for Statistical Computing, Vienna, Austria) and SPSS version 28.0 (IBM SPSS, Armonk, New York, USA). The level of significance for all analyses was α = 0.05.

### Role of the funding source

The funder of the study had no role in study design, data collection, data analysis, data interpretation, or writing of the report. All authors had access to the study data and reviewed and approved the final manuscript.

## Results

### Baseline characteristics

**Fig. 1:**
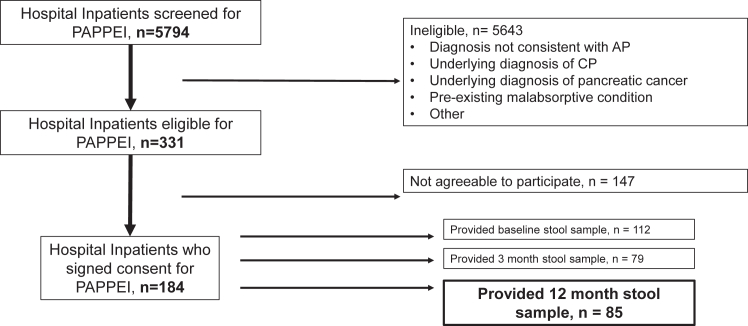
Flow diagram of study participation. PAPPEI: Post-acute pancreatitis pancreatic exocrine insufficiency study; AP: acute pancreatitis; CP: chronic pancreatitis.

A total of 85 participants completed assessment for EPI by submitting stool samples at 12 months after enrollment and were selected for analysis of the primary endpoint. Participants were recruited between June 22, 2017 and October 18, 2021. Of the approximately 5800 individuals who were screened for the study, 311 were eligible (see Fig. 1). Of the 184 who were consented, there were 112 who completed stool samples at baseline (mean 15 ± 13 days from pain onset to baseline stool collection), 79 who completed stool samples at 3 months; 64 participants completed stool samples at all timepoints. The majority of participants in the study population were white, obese, had gallstone etiology and mild severity of AP (Table 1). Most (∼65%) of participants were hospitalised with their index (first) attack of AP. Diabetes was present in 29 (40.8%) and EPI was present in 29 (40.8%) participants at the time of enrollment.

**Table 1:** Baseline demographic and disease characteristics by EPI outcome at 12 months.

	Level	Total participants (n = 85) N (% of total)	EPI (n = 29)	No EPI (n = 56)	p-value
Mean Age, years (SD)		54.7 (14.1)	55.8 (14.3)	54.1 (14.1)	0.59
Sex	Male	44 (51.8)	19 (65.5)	25 (44.6)	0.068
	Female	41 (48.2)	10 (34.5)	31 (55.4)	
Race	White	79 (92.9)	25 (86.2)	54 (96.4)	0.22
	Black	3 (3.5)	2 (6.9)	1 (1.8)	
	Multiple, other, declined to respond	3 (3.5)	2 (6.9)	1 (1.8)	
BMI	Non-Obese	39 (45.9)	15 (51.7)	24 (42.9)	0.44
	Obese (≥30 kg/m^2^)	46 (54.1)	14 (48.3)	32 (57.1)	
Etiology	Gallstones	33 (38.8)	4 (13.8)	29 (51.8)	0.018
	Alcoholic	9 (10.6)	4 (13.8)	5 (8.9)	
	Idiopathic	25 (29.4)	14 (48.3)	11 (19.6)	
	Hypertriglyceridemia	7 (8.2)	7 (6.9)	5 (8.9)	
	Post-ERCP	6 (7.1)	3 (10.3)	3 (5.4)	
	Other	5 (5.9)	2 (6.9)	3 (5.4)	
History of AP	First episode	55 (64.7)	13 (44.8)	42 (75.0)	0.0064
	Recurrent episode	30 (35.3)	16 (55.2)	14 (25.0)	
Smoking	Never	46 (54.8)	13 (46.4)	33 (58.9)	0.54
	Active	11 (13.1)	4 (14.3)	7 (12.5)	
	Former	27 (32.1)	11 (39.3)	16 (28.6)	
Alcohol	Never	24 (28.2)	8 (27.6)	16 (28.6)	0.78
	Active	37 (43.5)	14 (48.3)	23 (41.1)	
	Former	24 (28.2)	7 (24.1)	17 (30.4)	
Charlson Comorbidity Index	Mean (SD)	3.0 (1.7)	3.4 (1.9)	2.9 (1.6)	0.17
Pre-existing Diabetes	Yes	25 (29.4)	13 (44.8)	12 (21.4)	0.025
	No	60 (70.6)	16 (55.2)	44 (78.6)	
EPI at baseline	Yes	29 (40.8)	19 (82.6)	10 (20.8)	<0.0001
	No	42 (59.2)	4 (17.4)	38 (79.2)	
SIRS during admission	Yes	8 (9.9)	3 (10.7)	5 (9.4)	0.85
	No	73 (90.1)	25 (89.3)	48 (90.6)	
Severity of AP	Mild	52 (61.2)	12 (41.4)	40 (71.4)	0.017
	Moderately severe	25 (29.4)	14 (48.3)	11 (19.6)	
	Severe	8 (9.4)	3 (10.3)	5 (8.9)	
Necrosis	Yes	17 (27.4)	9 (40.9)	8 (20.0)	0.077
	No	45 (72.6)	13 (59.1)	32 (80.0)	

Data are presented as n (%) with percentages representing column values.

EPI: Exocrine Pancreatic Insufficiency; SD: Standard Deviation; BMI: Body Mass Index; ERCP: Endoscopic Retrograde Cholangiopancreatography; AP: Acute Pancreatitis; EPI: Exocrine Pancreatic Insufficiency; SIRS: Systemic Inflammatory Response Syndrome.

### Prevalence of EPI at 12 months following AP

EPI was present at 12 months in 29 (34.1%) participants. Of the participants with EPI, 19/85 (22.4% of the overall cohort) had severe EPI and 10/85 (11.8%) had mild EPI at 12 months after AP attack (Table 2). While the prevalence of severe EPI was higher in those with more severe grades of AP (30.3% for moderate/severe AP), even 12.8% of participants with an index, mild attack of AP (n = 39) had severe EPI at 12 months.

**Table 2:** Prevalence of EPI at 12 months amongst groups of interest within the study population (n = 85) with stool samples at 12 months.

Group	N	EPI	Mild EPI	Severe EPI
All	85	29 (34.1%)	10 (11.8%)	19 (22.4%)
Index AP	55	13 (23.6%)	4 (7.3%)	9 (16.4%)
Mild AP	52	12 (23.1%)	3 (5.8%)	9 (17.3%)
Index, Mild AP	39	5 (12.8%)	0 (0%)	5 (12.8%)

AP, Acute Pancreatitis; EPI, Exocrine Pancreatic Insufficiency.

### Predictors of EPI at 12 months

Compared to those participants without EPI at 12 months, a higher proportion of those with EPI had a history of AP prior to enrollment, had pre-existing diabetes, and had EPI based on FE-1 during enrollment (Table 2). The distribution of AP etiologies was significantly different between participants with and without EPI at 12 months, with a higher percentage of idiopathic, post-ERCP, and alcohol etiology seen in those with EPI and a higher percentage of gallstone AP seen in those without EPI (p = 0.018). More moderately severe and severe AP cases were seen in participants with EPI compared to those without EPI. Pancreatic necrosis also demonstrated a notable relationship with EPI (40.9% versus 20% for participants without EPI, p = 0.077; Table 1).

Based on the univariable results, history of AP, AP severity, pre-existing diabetes, and AP etiology (idiopathic versus non-idiopathic) were included in a logistic regression model to predict EPI at 12 months. Baseline EPI, although significant in the univariable analysis, was not included in the model to assess the practical prediction of EPI with non-stool-based measures. History of AP was not a significant predictor of EPI in the multivariable model, and the resulting parsimonious model included AP severity, pre-existing diabetes, and AP etiology. The odds of having EPI at 12 months were increased 4-fold with idiopathic AP etiology compared to non-idiopathic etiology, over 3-fold with moderately severe or severe AP compared to mild AP, and over 3-fold with baseline diabetes mellitus (Table 3).

**Table 3:** Multivariable logistic regression results for EPI at 12 months.

	β	SE(β)	OR (95% CI)	p-value
Constant	−2.02	0.47	0.13	<0.0001
Idiopathic Etiology	1.41	0.54	4.10 (1.42, 11.83)	0.0094
Severe/Moderate AP	1.15	0.51	3.17 (1.16, 8.67)	0.025
Pre-existing diabetes	1.17	0.54	3.22 (1.11, 9.30)	0.031

AP, Acute Pancreatitis; EPI, Exocrine Pancreatic Insufficiency.

### Natural course of EPI over 12 months following AP

**Fig. 2:**
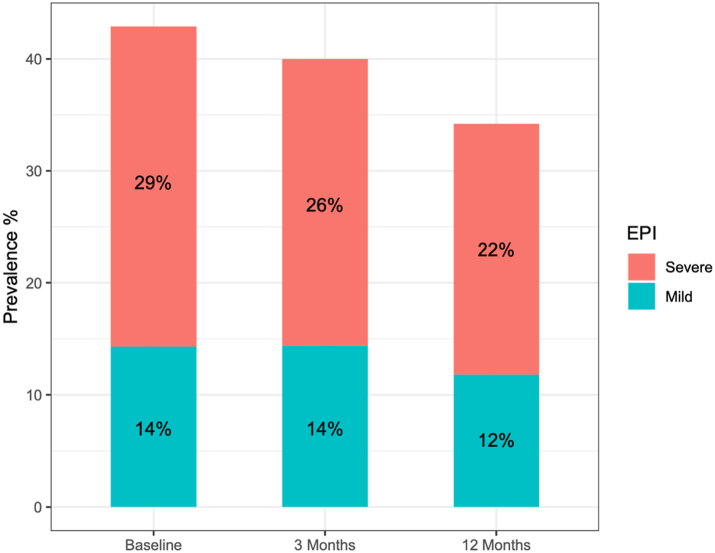
Prevalence of mild and severe exocrine pancreatic insufficiency (EPI) in participants with stool samples available at baseline (n = 112), 3 months (n = 90), and 12 months (n = 85) after AP (Fecal Elastase-1 [FE-1] level between 100 and 200 μg/g stool: mild EPI, and FE-1 level ≤100 μg/g stool: severe EPI).

The frequency of EPI after AP was lower at 12 months following AP attack; going from 43% of participants at baseline (n = 112) to 34% of participants at 12 months (n = 85). This was paralleled by a decrease in the prevalence of severe EPI from 29% at baseline to 22% at 12 months. In comparison, the proportion of participants with mild EPI stayed approximately the same over that time period (14% versus 12%; Fig. 2). Only 6 of the 85 participants who provided stool samples at 12 months reported having been initiated on PERT therapy over the course of the study. Of these, 3 were noted to have had EPI at baseline and 5 of these had EPI at 3 months, all data which was provided to the participant's primary care provider or gastroenterologist to decide whether to respond by initiating treatment.

**Fig. 3:**
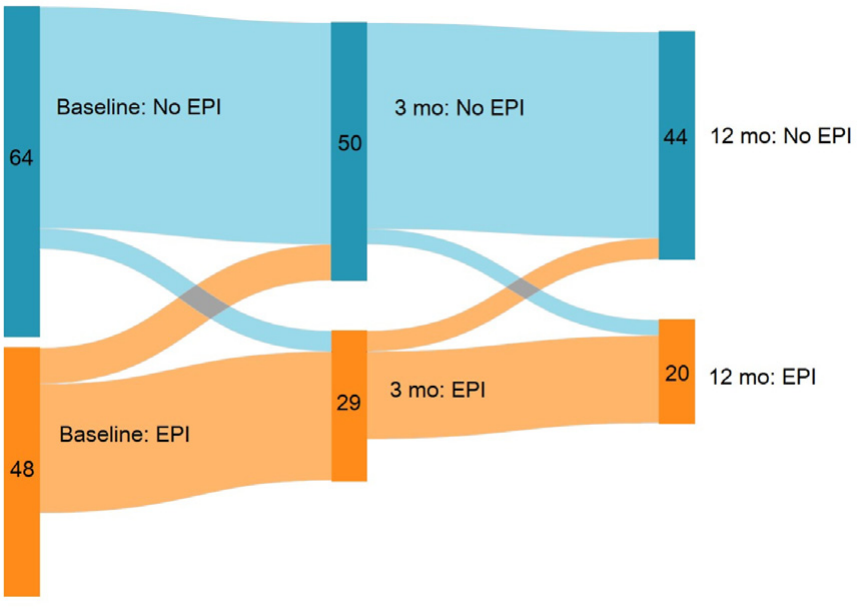
**Dynamic Natural History of EPI as diagnosed by Fecal Elastase-1 over 12 months following AP**. The diagram depicts the number participants with and without EPI during study follow-up amongst those with EPI assessment at baseline (vertical band at left n = 112) and at both 3 and 12 months (colored waves and bands to right). Participants who did not provide stool samples at either 3 or 12 months are not included in this diagram.

The natural history of EPI was examined in an analysis of participants who provided stool samples at baseline and all subsequent timepoints (n = 64; Fig. 3). A change in EPI status over this time period was seen in approximately 10% of participants who had baseline samples; the remainder stayed within the same category. Of the 48 with EPI at baseline, approximately 25% recovered over the subsequent 12 months: 7 recovered by 3 months but one redeveloped EPI by 12 months. An additional 4 recovered by 12 months. New occurrence of EPI was seen in 4 participants between baseline and 3 months, and one additional participant between 3 and 12 months.

## Discussion

To our knowledge, this is the largest prospective study of EPI following AP to date. We found that over one third of participants have EPI at 12 months post-AP. Independent predictors for the presence of EPI at 12 months included idiopathic AP etiology, moderately severe or severe AP, and diabetes mellitus at baseline. Of importance, EPI was diagnosed not only in participants with recurrent attacks or moderately severe/severe AP, but also following an index mild AP episode; albeit, with lower prevalence (13%).

The pathophysiologic processes that result in EPI after AP are largely unexplored and additional data are needed to understand the factors that lead to this phenomenon. One proposed idea is that the functions of pancreatic acinar cells are interrupted by the presence of inflammation, resulting in a ‘stunned’ phase that then resolves with waning of inflammation.^16^ This theory is supported by the incidence of EPI after index episode of AP seen in multiple studies including this prospective evaluation (highlighted in Table 2)—especially in those with only mild disease.^3^^,^^4^ Both the gradual development of EPI over the 12 months subsequent to AP episode and the lack of recovery seen in some participants (even with resolution of their inflammation; patterns such as those seen in Fig. 3) together suggest there are other contributing factors. These may include direct injury to the acinar cells or ducts in the setting of necrosis, systemic inflammation which may take longer to resolve, or immune factors (local or systemic) resulting in altered signaling or decreased function of the acinar cells. To this point, in those participants with pancreatic necrosis in our study, rates of EPI after AP at 12 months were approximately double compared to those without (40.9% versus 20.0%), though this did not reach statistical significance in univariate analysis (p = 0.077) nor was it an independent predictor of EPI at 12 months after AP. Prior published data have shown association between necrosis and development of EPI after AP at later timepoints (mean of 36 months), again raising the dynamic nature of EPI recovery (and potential for our study limited to 12 months after AP episode to miss a later recovery) as an additional aspect of understanding this prospective data in appropriate context.^4^

The severity of AP has previously been associated with development of EPI secondary to AP—both during hospital admission with the acute illness and in follow-up of varying lengths. In our study, moderately severe or severe AP was associated with an over 3-fold increase in the odds of developing EPI after AP at 12 months following an episode compared to mild AP. A systematic review and meta-analysis of EPI following AP found a two-fold increase in the prevalence of EPI in follow-up for participants who had severe AP compared to mild disease (30–40% versus 16–21%).^3^ Variable lengths of follow-up were completed in the meta-analysis, and multiple methods of testing (both indirect and direct testing) were used to diagnose EPI, limiting the ability to make direct comparisons. A prior meta-analysis evaluated the prevalence of EPI in participants with AP at a mean of 36 months after index admission limited to studies that employed FE-1 for diagnosis of EPI. In that analysis the prevalence of EPI after AP still remained higher in those with severe AP (33.4%) versus mild AP (19.4%).^4^ Again, the timing of follow-up in these participants may explain some of the variation in rates of EPI prevalence that are seen in comparison to our study, as EPI after AP has been previously shown to be dynamic with recovery in some participants increasing following a 36 month follow-up timepoint.^3^

Idiopathic etiology of AP in this study was independently associated with EPI at 12 months after AP, with an odds ratio of 4. Idiopathic AP encompasses those cases with no identifiable cause, which can occur in up to one third of cases.^17^ This may include participants who have genetic conditions not yet identified that predispose them to AP, biliary sludge that remains undetected on imaging, medication use that could have triggered an attack, or surreptitious alcohol use, but determination of idiopathic etiology overall requires the systematic exclusion of other potential causes, a process that is often performed at tertiary care centres. This multi-centre study included three high-volume tertiary care centres with extensive expertise in pancreatology where such patients are often referred, a potential source of explanation as to why an ‘idiopathic’ etiological category for AP was found to be a novel risk associated with EPI where it has not been for many other studies. Participants with an ‘idiopathic’ etiology of AP may have had an additional motivation to understand more about their disease leading to higher rates of participation. Furthermore, risk factors not yet understood in AP—a disease for which to date there still exists no cure and no disease-modifying pharmacologic treatment—are encompassed within the ‘idiopathic’ etiology, suggesting that herein lies an enriched population in whom additional investigations are needed.

AP etiology association with EPI in previous studies has largely focused on alcohol, and some studies have not reported idiopathic as a category, rather choosing to report this within ‘other’ or not at all.^4^ In the meta-analysis by Hollemans et al., EPI after AP was identified more frequently with alcohol etiology than in biliary etiology or ‘other’.^4^ In the meta-analysis performed by Huang et al., alcohol etiology was associated with a risk ratio of 1.6 for EPI after AP compared to gallstone etiology.^3^ The contribution of alcohol has been thought to be mainly secondary to the repeated injury suffered by the pancreas with continued alcohol use. This pattern is consistent with the increased risk of recurrent acute pancreatitis being higher in those with alcohol etiology than gallstone or other non-toxic etiologies.^18^ This is further supported by animal studies that suggest progression to EPI after AP due to alcohol even in the absence of the development of chronic pancreatitis.^19^ In our study, alcohol etiology was not significantly associated with higher risk of developing EPI after AP, however this finding may have been limited by the relatively low participation rate of patients with alcohol etiology of AP as they anticipated challenges with follow-up and declined participation at rates higher than those with other etiologies of AP.

Diabetes mellitus at baseline was also determined to be an independent predictor of EPI at 12 months after AP. In patients with chronic pancreatitis, the association between endocrine and exocrine insufficiency has been previously reported, with suggestion that beta-cell function and pancreatic enzyme secretion are strongly correlated.^20^^,^^21^ Lower C-peptide levels following stimulation with oral glucose and intravenous (pancreatic) secretagogues correlate with lower serum amylase and lipase levels, supporting a suspected mechanism of simultaneous damage to beta-cells and acinar cells from fibrosis that results in concomitant decrease in appropriate responses from each cell type.^21^ This mechanism, however, remains to be confirmed and much remains unknown about the role of exocrine-endocrine crosstalk of the pancreas in health and disease.^22^

In this study the determination of EPI was made by use of FE-1 stool testing, which represents the most commonly used and least burdensome objective test for the diagnosis of EPI. FE-1 has been developed and widely disseminated for the purpose of identifying EPI,^23^ but it has diagnostic limitations in the setting of watery stool from other causes,^24^ and inability to show response to PERT (it is unaffected by exogenous pancreatic enzyme supplementation). FE-1 has a moderate sensitivity between 54% and 75% in mild to moderate EPI,^25^^,^^26^ with a specificity at FE-1 level of <200 μg/g (commonly used for EPI in CP) of 63%.^27^ Recent estimates from nine studies using FE-1 in mild EPI revealed a pooled sensitivity of 49%; and from seven studies using FE-1 in moderate EPI a pooled sensitivity of 67%.^28^ While other EPI diagnostic tests are available, such as the coefficient of fat absorption or direct endoscopic pancreas function tests, their use is severely limited by the substantial burden placed on participants, high costs, and limited availability outside of specialty centres. The challenge of diagnosing EPI is a general limitation of this field at present and not unique to this study. In order to at least partially address this challenge and simultaneously acknowledge the unavoidable limitation regarding the significance of fecal elastase values between 100 and 200 μg/g stool, we have considered the prevalence of mild and severe EPI separately to allow readers to assess these findings independently (e.g., Fig. 2).

Our study represents a prospective assessment of EPI in a multicentre longitudinal cohort of patients following an AP episode. Our work aligns with a recent review^5^ that introduces the notion of providing long-term surveillance after hospital discharge for AP with the focus on early identification of new-onset diabetes and EPI. Specifically, the authors recommend FE-1 as the proposed test for EPI assessment to be performed initially after hospital discharge, then annually. Our study provides new high-quality evidence on the timeframe of an EPI surveillance program based on prospective data from three US sites. As the authors suggest more experience needs to be accumulated globally on the use of such surveillance programs and subsequently on the effects of interventions in long-term outcomes.^5^

There are numerous strengths of this study including its prospective, multicentre design, serial time intervals for stool collection, and follow-up for one year after the AP episode. However, there are also some limitations to acknowledge. Participants recruited at the three tertiary care centres were often transferred from outside hospitals where management techniques may differ more widely than in a tertiary care setting. This referral pattern may account for some degree of selection bias regarding AP severity and etiology. In addition, participants were informed prior to enrollment of the need for follow-up visits to complete the study activities; a lower rate of participation was seen therefore of individuals who anticipated challenges with follow-up. There was also a 24% drop-out rate between participants that provided stool samples on enrollment compared to the 12-month follow-up. While there are numerous factors that can account for this the study was also recruiting during the onset of the coronavirus pandemic of 2020: changes in the retention pattern may be a source of bias due to motivation on the part of those with symptoms at 12 months to continue participating. Due to the study design (and use of FE-1 for EPI diagnosis) we are unable to comment on the impact of treating EPI identified by a reduced FE-1 level: while the result of this specific biochemical test may be an indication for initiation of PERT therapy, the clinical manifestations of EPI have not been fully explored to date and require further work. Initiation of PERT was uncommon in this cohort (6/85, 7% with EPI at 12 months) despite notification of the participant's primary care physician or pancreatologist of abnormal FE-1 results suggesting this question will require a clinical trial to be answered. Factors affecting PERT initiation may include both extensive pill burden with which compliance is an issue, and PERT's high cost which can be unaffordable for many. While variable PERT exposure may have had an impact on recovery from symptoms of EPI, this would not confound our analyses due to lack of effect on FE-1. The final number of participants in this study totaled only 85: although this is the largest prospective study of EPI after AP to date, additional factors associated with development of EPI may not have been not detected here due to type II error. Although diagnosis of CP was a reason for exclusion from the study, lack of diagnostic clarity in the field on what constitutes early CP may result in patients with earlier phases of CP being included in this work; further studies are needed to understand what constitutes early CP and what role exocrine insufficiency may play in that diagnosis.

In conclusion, in this large prospective study of patients with AP, EPI was present in one-third of participants at 12 months after AP attack. Increased severity of AP, pre-existing diabetes, and idiopathic etiology were independently associated with EPI after AP. Importantly, EPI was also observed in >10% of patients with a mild, index episode of AP. Additional studies to follow the natural history of EPI beyond 12 months to assess its long-term implications as well as medical management after diagnosis are needed.

## Contributors

AEP (collection, analysis, and interpretation of data, drafting and critical review of manuscript), JB (collection and interpretation of data, critical review of manuscript), SC (study design and conception, analysis and interpretation of data, critical review of manuscript), JC (collection of data and critical review of manuscript), PJL (critical review of manuscript), JDM (critical review of manuscript, interpretation of data), VKS (critical review of manuscript), EA (critical review of manuscript), MLR (data collection, interpretation of data, review of manuscript), PP (data collection and interpretation, critical review of manuscript), KS (study design, data collection and interpretation), MN (data analysis and critical review of manuscript), PAH (data analysis and interpretation, drafting and critical review of manuscript), GIP (study design and conception, data analysis and interpretation, drafting and critical review of manuscript). AEP, SC, GIP have access to and verify the underlying study data. All authors approved the final version of the manuscript.

## Data sharing statement

The data from this study are not publicly available, however data sharing may be considered following publication upon reasonable written request to the authorship team. Requests will be considered on a case by case basis.

## Declaration of interests

VKS declares being a Consultant to Ariel Precision Medicine, Panafina, and Horizon Therapeutics; a Scientific Advisory Board Member of and equity holder in Kyttaro, Origin Endoscopy and Solv Endotherapy. SC declares funding for research, including salary, paid to her institution by Abbvie. MLR declares support from the Cystic Fibrosis Foundation and the American Society for Parenteral and Enteral Nutrition, and position as Chair for the Young Trainees Committee for the Collaborative Alliance for Pancreatic Education and Research (CAPER). PJL declares the Investigator Initiated Research funding from Abbvie for the project execution and administration. GIP declares the Investigator Initiated Research funding that supported this study from Abbvie. All other authors declare no financial or other conflicts of interest.
